# A deep neural network model of the primate superior colliculus for emotion recognition

**DOI:** 10.1098/rstb.2021.0512

**Published:** 2022-11-07

**Authors:** Carlos Andrés Méndez, Alessia Celeghin, Matteo Diano, Davide Orsenigo, Brian Ocak, Marco Tamietto

**Affiliations:** ^1^ Department of Psychology, University of Torino, Via Verdi 10, Torino 10124, Italy; ^2^ Section of Cognitive Neurophysiology and Imaging, National Institute of Mental Health, 49 Convent Drive, Bethesda, MD 20892, USA; ^3^ Department of Medical and Clinical Psychology, and CoRPS - Center of Research on Psychology in Somatic diseases, Tilburg University, PO Box 90153, 5000 LE Tilburg, The Netherlands

**Keywords:** artificial neural networks, emotion, superior colliculus, blindsight, deep learning, emotion recognition

## Abstract

Although sensory processing is pivotal to nearly every theory of emotion, the evaluation of the visual input as ‘emotional’ (e.g. a smile as signalling happiness) has been traditionally assumed to take place in supramodal ‘limbic’ brain regions. Accordingly, subcortical structures of ancient evolutionary origin that receive direct input from the retina, such as the superior colliculus (SC), are traditionally conceptualized as passive relay centres. However, mounting evidence suggests that the SC is endowed with the necessary infrastructure and computational capabilities for the innate recognition and initial categorization of emotionally salient features from retinal information. Here, we built a neurobiologically inspired convolutional deep neural network (DNN) model that approximates physiological, anatomical and connectional properties of the retino-collicular circuit. This enabled us to characterize and isolate the initial computations and discriminations that the DNN model of the SC can perform on facial expressions, based uniquely on the information it directly receives from the virtual retina. Trained to discriminate facial expressions of basic emotions, our model matches human error patterns and above chance, yet suboptimal, classification accuracy analogous to that reported in patients with V1 damage, who rely on retino-collicular pathways for non-conscious vision of emotional attributes. When presented with gratings of different spatial frequencies and orientations never ‘seen’ before, the SC model exhibits spontaneous tuning to low spatial frequencies and reduced orientation discrimination, as can be expected from the prevalence of the magnocellular (M) over parvocellular (P) projections. Likewise, face manipulation that biases processing towards the M or P pathway affects expression recognition in the SC model accordingly, an effect that dovetails with variations of activity in the human SC purposely measured with ultra-high field functional magnetic resonance imaging. Lastly, the DNN generates saliency maps and extracts visual features, demonstrating that certain face parts, like the mouth or the eyes, provide higher discriminative information than other parts as a function of emotional expressions like happiness and sadness. The present findings support the contention that the SC possesses the necessary infrastructure to analyse the visual features that define facial emotional stimuli also without additional processing stages in the visual cortex or in ‘limbic’ areas.

This article is part of the theme issue ‘Cracking the laugh code: laughter through the lens of biology, psychology and neuroscience’.

## 1. Introduction

Human and non-human primates excel in decoding emotional facial expressions, an ability fundamental for effective social interactions and linked to situations ancestrally relevant for survival [1–3]. This quick categorization of facial expressions relies heavily on distinctive and innate visual features that represent salient cues and guide attention. For example, we interpret lifted corners of the mouth as a smile, which communicates amusement, affiliation and bonding motivation. Fear is conveyed by the exposure of eye whites and indicates environmental danger, while sadness is channelled through angulate eyebrows and lowered lip corners [4–7].

Although sensory processing is pivotal to nearly every theory of emotion, the evaluation of the visual input as ‘emotional’ has been traditionally assumed to take place in supramodal brain regions, downstream to early areas directly involved in the initial analysis of sensory input [8–10]. For example, recognition of smiling and laughter engages the pregenual sectors of the anterior cingulate cortex [11,12]. Detection of danger and fearful expressions involves the amygdala, which also responds to other negative expressions such as sadness and anger [13–15]. According to this prevalent view, enhanced activity in the visual cortex to affective stimuli has often been interpreted as consequential, rather than integral, to initial emotional appraisal [16]. Additionally, subcortical structures receiving direct input from the retina, such as the superior colliculus (SC), the lateral geniculate nucleus or the pulvinar (pulv), are traditionally conceptualized as passive relay centres that transmit visual information, void of emotional meaning, to the visual cortex or other ‘emotional’ structures that conduct a value-based appraisal [17].

Recent literature challenges this traditional account and reveals a role for subcortical visual structures in more complex analyses of retinal input than previously believed [18]. Specifically, the SC, a laminated structure located astride the dorsal surface of the midbrain, seems endowed with the necessary infrastructure and computational capabilities for the innate recognition and initial categorization of emotionally salient features from retinal information [19–22]. First, response selectivity in the SC to face-like patterns or to stimuli evolutionary relevant for survival (e.g. prey, predators, food) occurs as early as 50 ms after the stimulus onset [23–25]. For comparison, the human amygdala's shortest responses to facial expressions have been reported at about 70 ms, while emotional modulation of V1/V2 activity peaks at 80 ms [26]. Second, experimental interference with V1 activity (e.g. visual masking, flash suppression, transcranial magnetic stimulation), or its permanent damage in patients with V1 lesion and cortical blindness, does not abolish non-conscious emotion recognition (affective blindsight) [27–30]. Affective blindsight patients tested across different studies attain on average 70% expression discrimination accuracy in 2-alternatives forced choice tasks (2-AFC; chance level = 50%), compared to nearly perfect accuracy in healthy observers under the same conditions, or greater than 80% proficiency in 6- or 7-AFC (chance level = 14%). These behavioural findings are complemented by neuroimaging experiments demonstrating that V1-independent encoding of emotional expressions is associated with selective activity in extra-geniculate pathways, of which the SC is the first and primary recipient of retinal information [31–36]. Third, the necessary contribution of the SC in the initial evaluation of facial expressions is also suggested by the consequences of manipulating low-level image features towards properties to which the SC is tuned to respond. These consequences are inherited from the nature of its retinal input, which arises primarily from the magnocellular (M) (and koniocellular—K) pathway, compared to little or no projections from the parvocellular (P) pathway. The M pathway is largely insensitive to colour, has a high sensitivity to minor luminance differences, and can resolve low spatial frequencies (LSFs). By contrast, the P pathway is attuned to colour in the green-to-red range and can resolve higher spatial frequencies [37]. Accordingly, neuroimaging evidence indicates that activity in the SC and structures receiving its ascending projections is constrained when faces are psychophysically biased towards low-level properties engaging the M pathway, such as small luminance variations, medium or long wavelengths and LSFs [38–42].

To better understand principles of emotion recognition, it is therefore of the utmost interest to characterize and isolate the initial computations and discriminations that the SC can perform on facial expressions, based uniquely on the information it directly receives from the retinal ganglion cells. Yet this evidence remains elusive owing to methodological limitations in animal neurophysiology and human neuroimaging. A promising new framework to explore these questions and complement neurophysiological investigation is emerging from the interaction between artificial intelligence and neuroscience [43–46]. Deep neural networks (DNNs) are computational models that potentially bridge the gap between cognitive functions and neurobiology. In fact, DNNs approximate how complex information-processing functions, such as visual recognition and categorization, may be carried out by biological neural networks. They consist of many processing units akin to neurons, arranged in interconnected layers analogous to brain areas, and with connections defined by weights that mimic the integration and activation properties of synapses [43,47–49]. DNNs learn through training to perform real-world tasks that essentially consist of mapping input patterns (e.g. raw images) to output classifications (e.g. sorting natural images according to categories like faces, objects and animals).

Current DNN models of the primary visual system attain human-like performance, predict representational transformations and reflect organizing principles of the primate vision (e.g. fine-to-coarse retinotopy, hierarchy, increasing perceptual invariance) [44,48,50]. However, these applications have been essentially grounded on models of the ventral cortical stream, starting from V1 and progressing to the temporal lobe, and there is no attempt to implement the architecture and constraints of the extra-geniculate visual system [51]. Here, we built a neurobiologically inspired convolutional DNN that approximates physiological, anatomical and connectional properties of the retino-collicular circuit. The DNN model incorporates a description of retinal output to the SC from different classes of ganglion cells forming the M, P and K channels, their relative proportions and terminal sites to the three different layers composing the superficial SC, as well as the internal architecture and weighted inter-layer connections [21,52,53]. Moreover, the model generates saliency maps whose function is to select conspicuous image locations for attentional selection and further analyses [54,55].

Trained to discriminate facial expressions of basic emotions and then asked to classify neutral, happy and sad expressions, our model reproduces above chance, yet suboptimal, classification accuracy, analogous to that reported in patients with V1 damage, hence matching human error patterns from V1-independent vision. When presented with gratings of different spatial frequencies and orientations never ‘seen’ before, the SC model exhibits spontaneous tuning to LSFs and reduced orientation discrimination, as can be expected from the prevalence of the M (and K) projections over the P channel. This response pattern is not found when the same stimuli are presented to DNNs that instead reproduce geniculo-striate (V1) architecture, which has a more balanced input from M and P channels. Likewise, face manipulation that biases processing towards the M or P pathway affects expression recognition in the SC model accordingly, an effect that dovetails with variations of activity in the human SC purposely measured with ultra-high field functional magnetic resonance imaging (fMRI). Lastly, differential extraction of salient face features as a function of the expressions arguably reflects functions and properties that indwell the retino-collicular pathway, as currently reported in human and non-human primates.

## 2. Methods

### (a) Model overview

**Figure 1.**
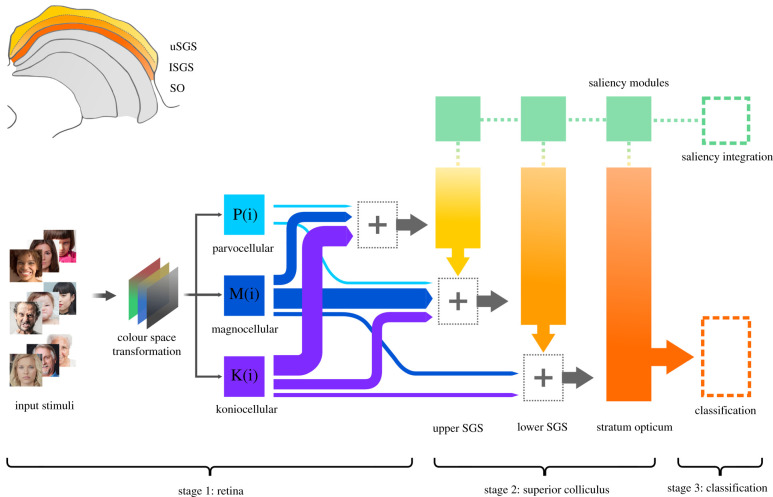
General overview of the model. In the upper left corner, an anatomical diagram of the SC, viewed from a coronal section, highlights the relevant superficial layers that have been modelled with corresponding layers in the DNN. Stage 1: input stimuli are colour-transformed and processed. Each one of the three main P, M and K channels is modelled by a function and the visual information is projected in different proportions to the appropriate SC layers. The varying width of the arrows represent the proportional contribution of each pathway to the corresponding superficial SC layer. A more thorough description of this stage is shown in figure 2. Stage 2: the three layers composing the superficial SC are modelled with fractal convolutional blocks and saliency modules that guide attention to select image regions for further analysis. Stage 3: after the final SC layer, the network uses a global averaging stage before a classification layer, while the saliency masks are integrated and contribute to generate the final output of the network.

The model uses goal-oriented supervised DNN with an overall architecture designed to match the neurophysiological and structural characteristics of the retino-collicular network. At its core, the model is composed of three main stages (figure 1).

First, we designed a front-end that reproduces retinal functions and differentiates among encoding properties of the P, M and K pathways that originate from different classes of retinal ganglion cells [56,57]. Each channel analyses the visual input in parallel and approximates the physiological characteristics of P, M and K pathways in terms of colour opponency, spatial frequency sensitivity, receptive field dimensions and centre/surround relationships.

Second, the model reproduces the circuit-level architecture of the superficial SC, with three sequential computational blocks of interconnected layers, analogous to the three *strata* of the SC that receive direct retinal input: upper stratum griseum superficiale (uSGS), lower stratum griseum superficiale (lSGS) and stratum opticum (SO) [52]. The information composing the input tensor of a particular layer derives from two main sources: (i) the proportional connections that each SC *stratum* receives from retinal P, M and K channels; and (ii) the output resulting from the computations performed in the preceding layer. Each layer performs several operations on the input, such as filtering and convolution through distinct kernels, pooling, in which the responses of nearby units are aggregated, and normalization. Also, each layer incorporates a saliency module that builds up a topographical map to prioritize processing of conspicuous regions in the visual image that should be analysed in more detail. The output of one stage of operations is a nonlinear combination of the input received and is then passed on to the next layer.

Third, the representations that are learned by the SC are processed and forwarded to an objective function that specifies the goal of the system, in our case a discriminative classification of emotional facial expressions. During training, the strength of connections between units is learned by the system based on experience rather than handcrafted by the researcher. The behaviour of the system (i.e. the accuracy in sorting facial categories) is the joint product of the architecture, objective function, learning rule specified. Accuracy is in fact a metric that shows how correct the model was during classification, defined as the proportion of correct predictions.

### (b) Stage 1: retinal output to the superficial layers of the superior colliculus

**Figure 2.**
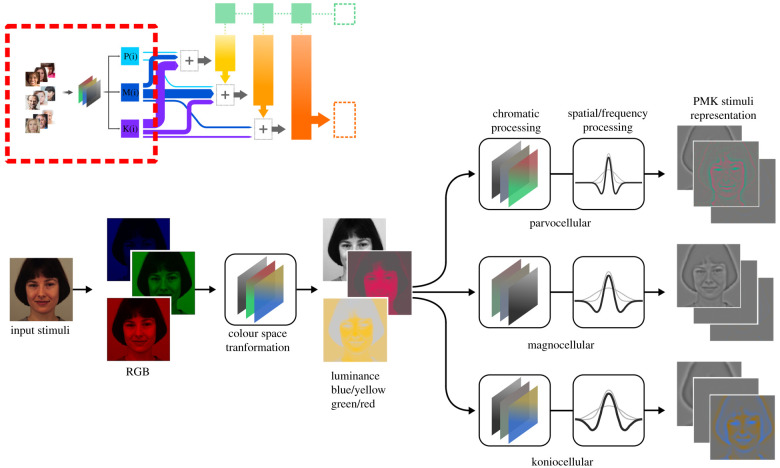
The processing of input stimuli by the three main retinal ganglion cells classes. The upper image inset shows a miniature of figure 1, where the area delimited by a red rectangle is expanded in the main image. The RGB representation of the input is converted to opponent colour space (luminance, red/green, blue/yellow). Subsequently, three distinct functions are applied in parallel, which model the individual chromatic responses and excitatory-inhibitory spatial characteristics of the P, M and K pathways. The resultant PMK representations are then directed to the rest of the SC model, as explained in the main text.

More than 20 morphologically distinct retinal ganglion cells have been reported and classically grouped in three distinct parallel channels—called P, M and K in primates—based on morphological, physiological and neurochemical distinctions [58–64]. In the present model, visual stimuli are initially processed in parallel by distinct functions that reproduce the encoding properties of P, M and K channels based on neurophysiological evidence. Specifically, the functions reproduce two distinct dimensions: (i) responses in the chromatic space; and (ii) inhibitory/excitatory spatial properties (figure 2). First, the input image is encoded in a typical long (L), medium (M) and short (S) wavelength (or red, green and blue—RGB) additive chromatic representation transformed into a colour-opponent space with three orthogonal cardinal axes: luminance, green/red and blue/yellow. After the chromatic space transformation, each P, M and K function is weighted to reproduce the chromatic opponency of each ganglion cell class: a dominant red/green response for the P channel (i.e. L − M), a mostly achromatic luminance response lacking colour opponency for the M channel (i.e. L + M), and a dominant blue/yellow response for the K channel (i.e. S − (L + M)) [56,65].

The second part of the P, M and K functions involves the mathematical characterization of the spatial and feature-processing properties distinctive for the three ganglion classes. Compared to P neurons, M cells have higher contrast sensitivity, lower spatial frequency selectivity, larger receptive fields and nonlinear neural summation at higher spatial frequencies [61,66–71]. K neurons form a more heterogeneous class that share with M and P cells common principles of centre/surround organization but have distinctive response properties in acuity and contrast sensitivity [72]. Major differences are concerned with details of their receptive field structure and firing characteristics, with K cells displaying blue-on receptive fields and linear as well as nonlinear summation over their receptive field.

Difference of Gaussian functions (DoG) is an established method for modelling receptive fields and the centre/surround relationships in retinal ganglion cells with a mathematically separable and easily computable approximation to the Laplacian of a Gaussian [73,74]. Moreover, it has the desirable property of expressing the main receptive field parameters simply as excitatory and inhibitory terms directly related to the underlying physiology.

The excitatory (*e*) and inhibitory (*i*) parameters of the DoG functions, expressed in degrees of visual angle, are as follows: P: $\sigma_{e}=0.12^{\circ },\;\sigma_{i}=0.56^{\circ }$, M: $\sigma_{e}=0.18^{\circ },\;\sigma_{i}=0.77^{\circ }$ and K: $\sigma_{e}=0.31^{\circ },\;\sigma_{i}=0.91^{\circ }$ [67,75].

With our stimulus dimensions and retinal set-up, one degree of visual angle equals 5.33 pixels, which, therefore, translates the above parameters into: P: $\sigma_{e}=0.64\mathrm{px},\;\sigma_{i}=2.98\mathrm{px}$, M: $\sigma_{e}=0.96\mathrm{px},\;\sigma_{i}=4.10\mathrm{px}$ and K: $\sigma_{e}=1.65\mathrm{px},\;\sigma_{i}=4.85\mathrm{px}$.

The P, M and K channels do not project uniformly to the different superficial *strata* of the SC. Overall, most retinal output to the superficial SC originates from M and K channels with a ratio of about 1.5 : 1, and only sparse projections come from the P channel [63,72,76,77]. Concerning intra-layer differentiation, the K channel projects more to the uSGS than to the lSGS, while M channels terminate chiefly in lSGS [52]. Each P, M and K tensor has been convolved with a kernel $K\;:\;R^{3}\to R^{N}$, in which the dimension *N*, of each feature map tensor derived from P, M and K functions has been weighted proportionally to reproduce the specific retinal output to different SC layers. Formally, the input tensor *i* to layer *l* is given by

2.1 $$i_{l}=o_{l-1}∥K_{\;p_{l}}\;*\;P∥K_{m_{l}}\;*\;M∥K_{k_{l}}\;*\;K,$$

where *o* is the output tensor of layer *l − 1*, while || and * are the concatenation and convolution operation. $K_{\;pl},\;K_{ml},K_{kl}$ represent the kernels of layer *l* with which the P, M and K tensors are, respectively, convolved (denoted with the *p*, *m* and *k* subindices in the equation) (figure 1, stage 1).

### (c) Stage 2: layer structure and interconnections

In this stage, we modelled the structural and inter-layer connectional architecture of the superficial SC as a convolutional DNN taking its inputs from the retina. Our approach includes multiple information paths inside each layer to reflect the complex nonlinear and non-sequential configuration of neuronal connections in the SC. This principled perspective can be accomplished by choosing among a variety of convolutional layer designs that incorporate the controlled branching of information paths. For example, residual networks (ResNets) adopt parallel convolutions with different receptive fields [78] and skip connections that allow identity mappings [79]. However, simply skipping connections has inherent drawbacks, among which the inability of residual blocks to force the gradient through the network weights during the training phase. This phenomenon commonly leads to under-learning, with just a few blocks in the network learning useful representations [80].

**Figure 3.**
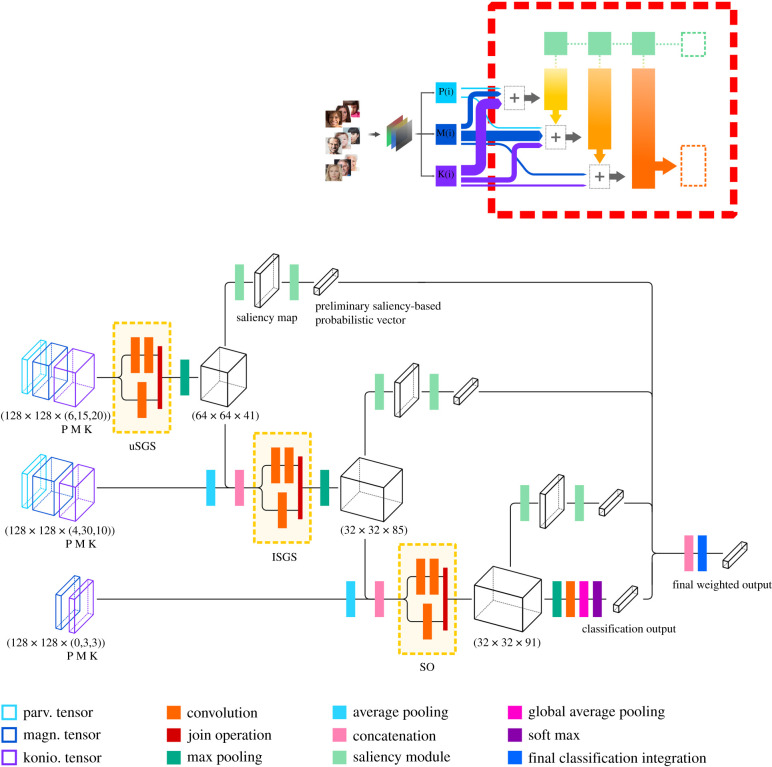
Detailed diagram of the model architecture. The upper image inset shows a miniature of figure 1, where the area delimited by a red rectangle is expanded in the main image. The tensors processed by the PMK functions serve as inputs to the SC layers, modelled with FractalNet blocks (yellow rectangles). The output of each SC layer contributes to the input tensor of the following layer. The tensor dimensions are specified in brackets. After the final SC layer, one feature map is generated for each category and global average pooling is performed on each of them instead of fully connected layers. The final output of the network is weighted by the saliency maps generated by modules operating on representations at each SC layer.

To derive more robust and flexible nonlinear functions, we, therefore, opted for FractalNet blocks among the available architectures that incorporate controlled branching of information paths [81]. As it happens, FractalNet possesses the following desirable properties. First, it has a modular convolutional architecture that does not rely on explicit residual connections, but nevertheless allows for a principled way to incorporate many paths in each modelled layer. Second, FractalNet blocks proliferate information paths in a symmetrically repeating architectural pattern, and the creation of multiple paths for the flow of information enables a form of deep regularization. Third, FractalNets are built on an expansion rule that creates truncated fractals with the form of parallel interconnected branches with a different number of convolutional layers, which allows the existence of multiple shortcut paths for flexible information flow. Formally:

2.2 $$F_{C+1}(z)=[\left(F_{C}\circ F_{C})(z\right)]⊕[K\;*\;z],$$

where *C* stands for the branch or column index of the truncated fractal in the FractalNet block $F_{C}$, ∘ and ⊕ correspond to the composition and join operation, respectively. In a FractalNet, the initial basic block $F_{1}(z)$ is a single convolution operation (K ∗ z) between a kernel *K* with a tensor *z*, which serve as the basis for the composition of the block. The number of convolutional layers on any given branch in the FractalNet block depends on the column index, with their number given by $2^{C-1}$. In our model, every SC layer is modelled by a fractal block with C=2; i.e. with just two parallel columns, allowing alternative data paths in each layer, and with rectified linear unit (ReLU) as activation function (figure 3).

The join operation is implemented as a layer performing element-wise averaging of multiple branches output, either with partial outputs along the block, or as a final operation joining all the column tensors at the end of the block.

To warrant the creation of relevant internal subnetworks with variable depths and discourage co-adaptation of sub-paths, FractalNet is trained with a regularization technique called drop-path. Drop-path prevents co-adaptation of parallel paths by randomly removing branches during each training iteration, as if that path were not a part of the network architecture. This is analogous to the way the dropout method removes regular neuronal units on standard feed-forward networks [82]. It allows for a variable set of architectural information paths to follow during training, while making the complete trained structure available at inference time. Drop-path guarantees that at least one path from input to output exists at each training iteration. It alternates between two sampling strategies: a local one, where branches are probabilistically dropped at each join layer, and a global approach, in which a single column is left standing between the input and output of the block, incentivizing the development of independent predictors.

Lastly, neurons in the superficial layers of the SC have been recently reported to encode a topographic saliency map of the visual scene that is unlikely to derive from the fronto-parietal cortex or pulv [54,83]. The function of the saliency map is to guide spatial attention for selection and further processing of the most informative image parts. Computational saliency modules have been previously built from architectures inspired by cortical organization, but never adapted or integrated with representations generated by a DNN of the SC [84,85]. To incorporate this function in our model, we modified a modular network architecture originally designed as an attention mechanism in fine-grained recognition [84]. Briefly, the architecture of our saliency modules consists of three main components: (i) the first component processes the feature maps generated at each SC layer and defines relevant spatial locations; (ii) the second component puts in competition for saliency different spatial locations within each map and generates a hypothesis based on saliency information; and (iii) the third one combines all feature maps fed in a bottom-up manner and generates a summary confidence score for each attentional block.

This process is applied to each of the three layers corresponding to the three SC strata. Then, the model learns to correct the initial predictions capitalizing on information from the SC layer modules. Finally, the module represents a complete account of bottom-up saliency processing endowed with internal dynamics, which generates attentional guidance to the most informative image locations (see the electronic supplementary material for details).

### (d) Stage 3: representation integration and emotion categorization

In typical CNN designs, the feature map tensor resulting from the last convolutional layer is transformed into a vector and its elements connected to fully connected layers, which ultimately serves as a classifier readout. This strategy has proven effective from a purely engineering perspective, but the solution creates an unnatural division of the model, because the convolutional layers are conceived purely as feature extractors that are conceptually separated from a subsequent classification stage. One additional problem concerns the interpretability and traceability of the decision information that propagates backwardly during training, because the fully connected layers at the classification stage essentially act as an inscrutable black box.

To circumvent these limitations and preserve an unbroken flow of information, which is more conducive for neurological modelling, we propose to modify the canonical CNN approach. The key change consists in the generation of one feature map after the last SC layer for each categorical classification, on which global average pooling is then performed. This operation combines all activations within each feature map and produces a scalar score per category next forwarded to a softmax layer that yields a classification probabilistic vector *o*_net_ [86]. This procedure preserves a natural flow of information across the whole model and promotes a continuous correspondence between the emergent representations in the SC layers and the emotional category each facial expression is supposed to belong to.

To obtain the final classification readout, the preliminary network prediction *o*_net_ is merged with the averaged output from the *S* saliency modules, i.e. the saliency prediction vectors $o^{l}\;:\;l\in \{1,\ldots ,S\}$, by means of a weighted sum, where a weighting parameter ω denotes the contribution of the main network classification *o*_net_:

2.3 $${\mathrm{output}}_{\mathrm{net}}=\omega o_{\mathrm{net}}+\frac{\left(1-\omega \right)}{\left|S\right|}\sum_{l\in 1\ldots S}o^{l}.$$

### (e) Implementation details

The model has been implemented in Tensorflow 1.13.1 and Keras 2.2.4 deep learning frameworks trained in a Linux PC with a Nvidia RTX 2070 GPU. From the comprehensive image dataset, the emotional categories of happiness, sadness and neutrality were selected, and the resulting dataset was divided into training and test datasets consisting of 20 689 and 750 images, respectively. The model was trained for 100 epochs with a batch size of 48 face images. Adam was used as a gradient descent optimization algorithm, and categorical cross entropy as a loss function [87]. Our model is initialized with a learning rate of 0.001, which is optimized by Adam during the learning procedure (see the electronic supplementary material, table S1 for the complete list of tensor dimensions and connections, and table S2 for model hyperparameters).

### (f) Model training and testing

#### (i) Image dataset and validation

An image web search engine was automatically queried with a sequential permutation of elements from a set of selected emotional terms with modifiers such as ethnicity, gender and age. Examples of the composite search strings are: ‘happy old man’, ‘crying child’ and ‘inexpressive white woman’. The resulting images were then processed by a face detection and selection algorithm. The automatic procedure centred the face and set the geometric boundaries of the image according to a pre-defined calculated field of view of 24°, while discarding the images that did not present at least one face. The pre-processed images were then thoroughly reviewed manually by researchers and the relevant ones carefully classified into one out of five pre-defined discrete categories facial expressions displaying happiness, sadness, anger, fear and emotional neutrality (26 914 total images, happiness: 16264, fear: 2531, anger: 2944, neutral: 2427, sadness: 2748). This approach enabled us to acquire a wide range of facial expressions with more variability, ethnicity diversification and ecological validity than a standardized dataset typically depicting front-view Caucasian faces.

To validate the image dataset, 207 subjects (mean age 27 years, s.d. = 9.22 years) were presented with a pseudo-random selection of 100 images balanced across emotional categories and asked to classify the stimuli in a 5-AFC without time limit. The protocol was programmed in psychopy v. 3.1.2 [88] and executed via the online experimental platform Pavlovia (https://pavlovia.org/). Average recognition accuracy was 0.77 (s.d. = 0.082). Overall, the consistency between intended expressions (i.e. as classified from web search and further scrutinized by the experimenters) and judged expressions (i.e. from test participants) was significant (Cohen *K* = 0.72; s.d. = 0.103). The extensive image dataset enabled system training and testing in quasi-naturalistic context with images in a wide range of real lighting conditions and without controlled backgrounds, thus composing multidimensional data that approximate real-life environments.

#### (ii) Recognition accuracy and generalization across datasets

After training, the accuracy of the retino-collicular network in the emotion categorization task was also tested with a different and independently validated dataset of facial expressions: the Karolinska Directed Emotional Faces (KDEF) [89]. This approach enabled us to assess the generalizability of our model's recognition accuracy over a broad and novel image context. Moreover, the co-registration property of the KDEF represents an opportunity to estimate average parameters of interest and nicely complements the variability of our own dataset with a standard and carefully controlled setup.

The KDEF is composed of 4900 pictures of facial expressions from 70 subjects displaying seven emotional categories from five different viewing angles. From the different angles, we selected the subset of front-facing images, for a total of 420 instances (140 images per each category: happiness, neutral and sadness).

#### (iii) Spontaneous emergence of spatial frequency tuning and orientation sensitivity

Here, we exploited the fact that the M, P and K pathways engage the SC differentially compared to V1 and ventral stream cortices. Consequently, SC's responses are tuned to different ranges of spatial frequencies and orientation discriminations that preferentially reflect the M pathway properties. In comparison, the geniculo-striate system has a more balanced contribution from M and P pathways and can, therefore, process more efficiently visual features analysed by either channel. If the retino-SC network is analogous to the real brain, then it should exhibit the same tuning properties, while differing from those displayed by DNNs that mimic the geniculo-striate system [90–92].

First, we analysed whether selective tuning to LSF and reduced orientation sensitivity emerge spontaneously as a function of the DNN architecture and without training the model for these specific properties. We presented the model with gratings of varying spatial frequencies and orientations never displayed during the learning phase, while activations of the last convolutional layers were recorded (spatial frequencies: from 0.16 cycles per degree to 2.19, at 0.053 steps; orientations: from 0°—vertical—to 90°—horizontal—, at 3° steps clockwise). Next, we investigated the responses to the same gratings presented to two additional DNN models, AlexNet and VGG19, designed and validated to approximate the biological structure and response properties of the geniculo-striate (V1) system [90–92]. The three DNNs were compared using representational similarity analysis, in which each model representations are compared at the level of the dissimilarity structure of their response patterns [93].

#### (iv) Face manipulation towards magnocellular and parvocellular channel properties

*Impact on DNN recognition accuracy.* Electrophysiological and fMRI studies in both healthy and V1-damaged patients consistently found a greater signal in the SC and connected structures (e.g. pulv and amygdala) to LSF (M-biased) than to high spatial frequency (HSF) faces (P-biased) [26,38,40]. To examine how spatial frequency manipulation impacts on emotion recognition in the DNN model, we filtered the KDEF faces with a Butterworth filter using a high-pass cut-off of 24 cycles per image for HSF (0.188 cycles per pixel or one cycle per degree), and a low-pass cut-off of six cycles per image for LSF stimuli (0.047 cycles per pixel or 0.25 cycles per degree), according to human literature parameters.

*Impact on fMRI activity in the human SC.* A complementary method to engage preferentially the M or P processing involves adjusting the luminance and colour of image stimuli. M-biased stimuli consist of achromatic/heteroluminant faces (i.e. greyscale and low-luminance contrast), whereas P-biased stimuli are heterochromatic/isoluminant images created by converting greyscale faces to individually calibrated isoluminant intensities of red and green [94,95]. M-biased faces have been proven to affect behavioural tasks engaging the SC (e.g. saccade frequency and latency) as well as reflexive amygdala responses to facial expressions [96,97], consistent with the notion of a subcortical pathway to the amygdala that facilitates rapid, but coarse, emotional processing [13]. Unlike spatial frequency manipulation, fMRI activity in the human SC to M- and P-biased faces derived from luminance and colour manipulation has never been studied directly.

To this end, fMRI response in the SC of two participants was acquired in an ultra-high field 7 T scanner while viewing, in a multiple block design, M- and P-biased neutral and fearful facial expressions from the KDEF dataset (see the electronic supplementary material for details). *Z*-scores of blood oxygen level-dependent beta in the SC for the experimental conditions were compared with robust Bayesian parameter estimation (RBPE) to yield the complete distribution of parameter values and estimate the posterior probability that these activities are credibly different [98].

#### (v) Other sources of image perturbation

It is commonly held that biological vision exhibits robustness and generalizability that is lacking in artificial neural networks. For example, image perturbation with the addition of uniform noise or salt-and-pepper (S&P) noise affects object recognition in an artificial neural network far more than in human observers [99,100]. However, there is no evidence on how these types of image perturbations interfere with the encoding properties of a network designed to reproduce the architecture of the retino-collicular system.

To explore these dimensions, the pattern of response in the DNN model was also tested with controlled image noise. The KDEF dataset was processed using a varied set of additive Gaussian noise with progressively larger standard deviations (from 5 to 75 s.d.). Values from a random Gaussian distribution have been added to the original images, with the additional noise approximating the effect of diverse random processes that occur in nature. Stimuli were also manipulated with a S&P degradation, which is a variable granular perturbation of the image pixels in the form of sudden impulses that lead the affected pixel to the maximum or minimum value of the image range. The stimuli were processed with different amounts of S&P noise, from 0.5% up to 10% of the total image pixels.

#### (vi) Feature extraction and saliency maps

*Occlusion technique*. In the real world, occlusion of facial parts is common and often arises from clothing or movements. It is well established that certain face parts, like the eyes or the mouth, provide higher discriminative information and saliency than other parts, such as chin, nose or ears when it comes to discriminating emotions [101]. Moreover, the face cues associated with better emotion recognition may vary depending on the specific expressions.

We implemented facial occlusion to investigate which facial parts are preferentially exploited by the model and compared the network performance with evidence on human observers. The occlusion sensitivity analysis consists of the systematic occlusion of limited portions of the input image with a grey patch and then relates the classification accuracy to the region of the face that has been removed [102].

*Bubbles technique*. The ‘Bubbles' technique essentially addresses the same question of the occlusion to reveal local expression-driven features important to distinguishing facial expressions, but the approach is reversed [5]. In this case, only limited portions of the face are revealed, by adding to the image random Gaussian windows with an iterative procedure, which is similar to viewing facial expressions through a cardboard poked with random little holes. The method is self-tuned, meaning that it adjusts the number of Gaussian bubbles in each iteration to keep a balance between acceptable accuracy and relative difficulty for the model across trials that compose the procedure.

**Figure 4.**
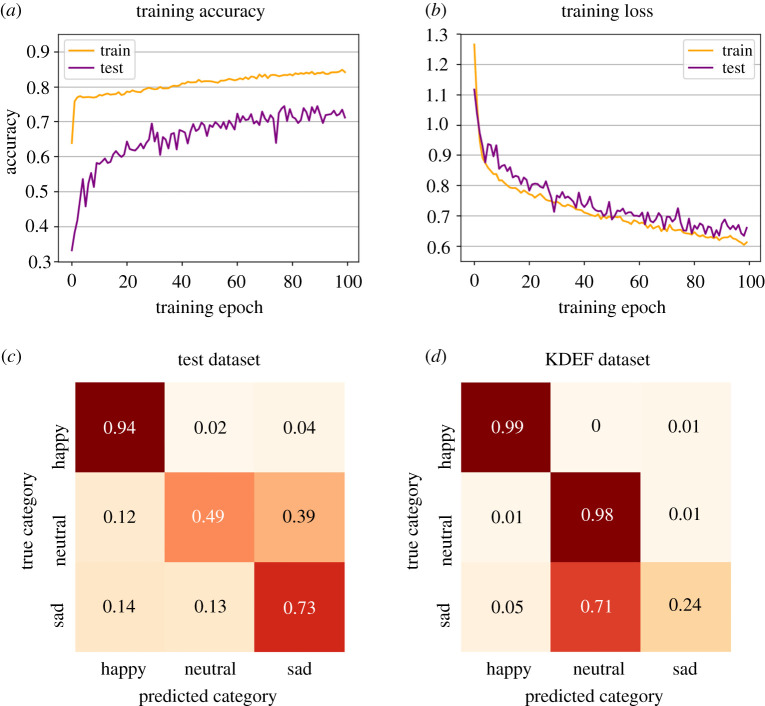
Learning performance and recognition accuracy. Accuracy (*a*) and loss learning curves (*b*) of the model evaluated with our training and testing datasets. The orange lines represent the learning phase, while the purple lines denote the evaluation performed with the test dataset at the end of every epoch. (*c*,*d*) Normalized confusion matrix describing the classification results with the test (*c*) and the KDEF dataset (*d*).

We used a bubble size with a standard deviation of 7% of stimulus size, which translates to $\sigma =1.68^{\circ }=8.96\mathrm{px}$, and 500 iterations per stimulus. Our Bubbles implementation was applied to the front-view KDEF faces. To summarize results in compact and easily accessible format, the averaged results were projected on the averaged KDEF (AKDEF) dataset, which is the average face image of the spatially registered KDEF dataset [103]. See figure 4 for examples.

## 3. Results

### (a) Learning performance, recognition accuracy, precision and sensitivity

Figure 4 shows the learning trajectory of the network model expressed through the evolution of overall accuracy and the minimization of loss values across 100 training epochs, where loss is defined as the value returned by the cost function of the network to be minimized during training. After training, accuracy in categorizing previously unseen happy, sad and neutral faces in the test dataset was above chance level (71%; chance = 33%). For comparison, recognition accuracy in healthy human observers confronted with similar facial expressions is typically between 80% and 90%, even though tasks are usually more demanding, with forced-choice alternatives varying between 6- and 7-AFC across ‘basic emotions' (chance level between 16.6% and 14.3%) (electronic supplementary material, tables S3 and S4) [104–108]. Notably, the model's accuracy, as emerging from its architecture, matches the one reported in patients with V1 damage who rely on extra-geniculate and retino-collicular pathways and attain recognition accuracy of around 70% [109–114].

The confusion matrices in figure 4*c*,*d* report a detailed description of categorization accuracy and errors for two different test datasets as a function of the three expressions. Measures of ‘precision’ and ‘sensitivity’ offer additional insights into the model's performance. Formally, model precision indicates the ratio of true positives over true plus false positives. Precision answers the following question: among facial expressions that the model categorizes as happy (or sad or neutral), how many faces are truly happy (or sad or neutral)? Sensitivity (or ‘recall’ in machine learning literature) is defined as the ratio of true positives over true positives plus false negatives. Sensitivity addresses this question: among all happy (or sad or neutral) facial expressions, how many instances have been correctly classified as happy (or sad or neural)? Finally, the F1 score is calculated as the harmonic mean between precision and sensitivity, thus summarizing these two measures with a single scalar.

When tested with our dataset, the model's precision was higher for happy (78%) and neutral (77%) expressions than for sad expressions (63%). Likewise, sensitivity shows a higher value for happy expressions (94%), followed by sad (73%) and neutral faces (49%) (F1 scores: happy = 0.86, neutral = 0.60, sad = 0.68). Therefore, like human observers, happiness was recognized more easily than other emotional expressions [115,116]. Finally, when the model was asked to discriminate KDEF faces, precision was 95% for happy faces, 92% for sad and 58% for neural expressions, whereas sensitivity was 99% and 98% for happy and sad expressions, respectively, but fell below chance for sadness (24%), (F1 scores: happy = 0.97, neutral = 0.73, sad = 0.38).

### (b) Emergence of spatial frequency tuning, orientation sensitivity and comparison with V1 models

The response patterns to a range of spatial frequencies and orientations in the SC model were qualitatively different to those emerging in V1 models, which in turn did not differ from each other.

**Figure 5.**
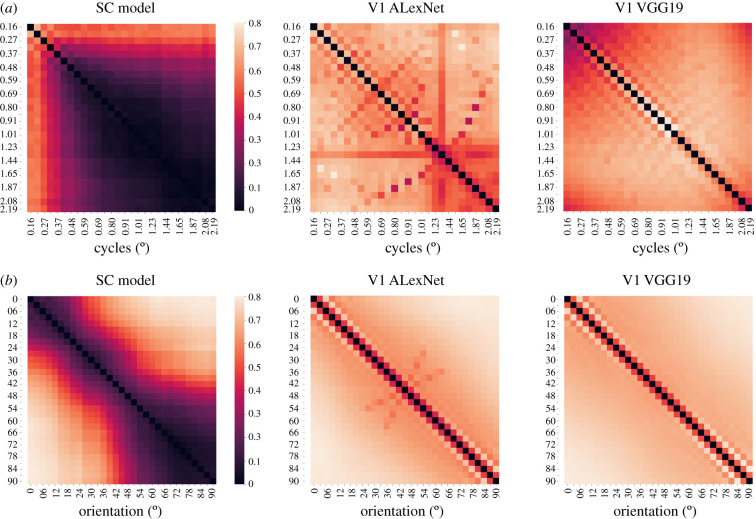
Spontaneous emergence of spatial frequency tuning and orientation sensitivity. (*a*) Discrimination performance across gratings of different spatial frequencies in the SC model and in the AlexNet and VGG19 models that approximate V1 responses. (*b*) Discrimination performance across gratings of different orientations in the SC model and in the AlexNet and VGG19 models that approximate V1 responses.

As shown in figure 5*a*, tuning to LSF spontaneously emerged in our artifical SC, as the DNN model could differentiate proficiently among gratings with different ranges of spatial frequencies, as long as they remained lower than 0.6 cycles per degree, but progressively reduced its discriminatory capability at increasing spatial frequencies. Notably, neuronal responses in the SC of non-human primates are typically tuned to LSF (less than 1.5 cycles per degree, with optimal responses at 0.56 cycles per degree) [117]. Likewise, optimal parameters for detection of static stimuli in patients with V1 damage lie in a similar range of LSF [118]. Both models of the geniculo-striate system showed nearly perfect responses, resolving gratings discrimination throughout the whole range of spatial frequencies, as can be expected from the integration of M and P channels in V1.

Concerning orientation, the SC model could spontaneously discriminate gratings that varied from 9° to 12°, whereas both V1 models showed differential activations for gratings with variations of 3° (figure 5*b*). These sensitivity differences are in accordance with the orientation discrimination reported in blindsight patients, who differentiate orientations varying of approximately 10°, as compared to 2–3° accuracy at the corresponding locations in the intact visual field [119].

### (c) Effects of face manipulation towards magnocellular and parvicellular properties

#### (i) Impact on deep neural network recognition accuracy

**Figure 6.**
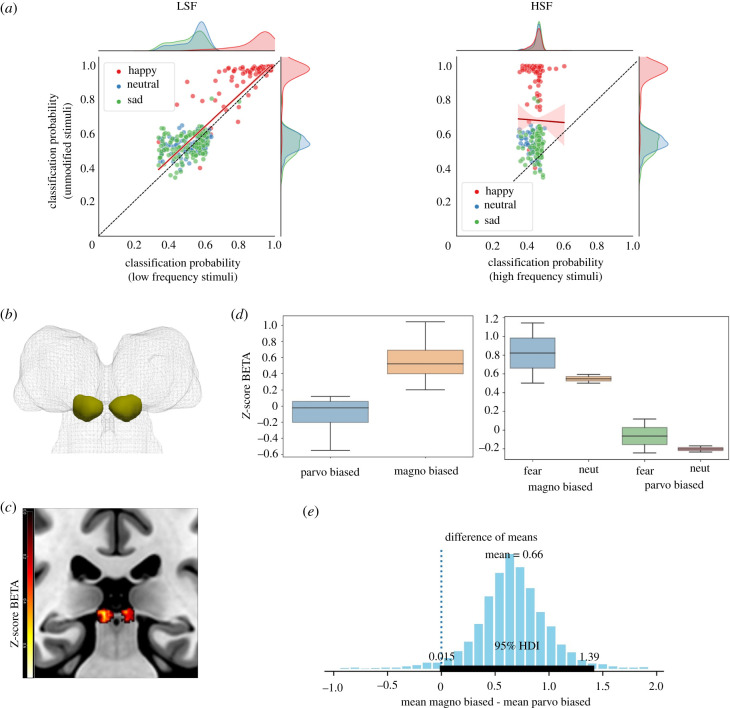
Effect of face manipulation towards M and P channels in the SC model and in the fMRI activity of the human SC. (*a*) Scatterplots and marginal distributions of classification probability for LSF and HSF filtered faces compared to original images. The *y*-axis corresponds to the probability of belonging to the correct category assigned by the model to each unfiltered image, while the *x*-axis represents the probability assigned when the instances are spatial frequency filtered. The red line and shaded area denote the best linear regression fit to the data and its 95% confidence interval. (*b*) Three-dimensional rendering of the anatomical definition of the SC. (*c*) Response of the SC across all experimental conditions expressed as *Z*-score beta. (*d*) Boxplot of parameter estimates showing increased response in the SC for M-biased than P-biased stimuli. (*e*) Posterior distribution from Bayesian estimation of the mean difference between fMRI activity induced by M- and P-biased faces (thick line on the *x*-axis denotes values that fall inside 95% highest density interval (HDI)).

In keeping with previous neuroimaging and electrophysiological assays, LSF faces did not impact on the model's discrimination performance, which maintained above-chance expression categorization and general accuracy almost identical to that yielded with unfiltered faces (68%). Conversely, classification dropped down to almost chance level with HSF faces (35%) (figure 6*a*).

#### (ii) Functional magnetic resonance imaging activity in the human superior colliculus

Signal intensity within the human SC of two subjects was greater for M-biased than P-biased face stimuli, irrespective of expressions (figure 6*b–d*). In keeping, the comparisons with RBPE between the activity induced by M- and P-biased stimuli revealed that the 95% highest density interval (HDI) of posterior probability of this difference was greater than 0 (mean difference = 0.66, 0.015 < HDI < 1.39) (figure 6*e*). There was also a trend towards higher activity for fearful than neutral expressions; however, the 95% HDI did not fall outside zero (mean difference = 0.0857, −1.26 < HDI < 1.6).

### (d) Gaussian and salt-and-pepper noise

The performance of the SC model progressively deteriorated as the input was degraded with the addition of Gaussian and S&P noise (electronic supplementary material, figure S2). Both image perturbations differentially affected discrimination of the three facial expressions. Recognition of sad faces was the most impaired and dropped at chance with the inclusion of Gaussian noise at approximately 15 s.d.s and with 2% S&P noise. Recognition of happy expressions was the least impaired, with a steep decrease of accuracy slope that stabilized at 30 s.d.s of Gaussian noise and 5% S&P noise. Lastly, the impact on sad expressions fell midway, with almost a linear decrease in the recognition of sad expressions with the progressive increase of image noise.

### (e) Feature extraction and saliency maps

**Figure 7.**
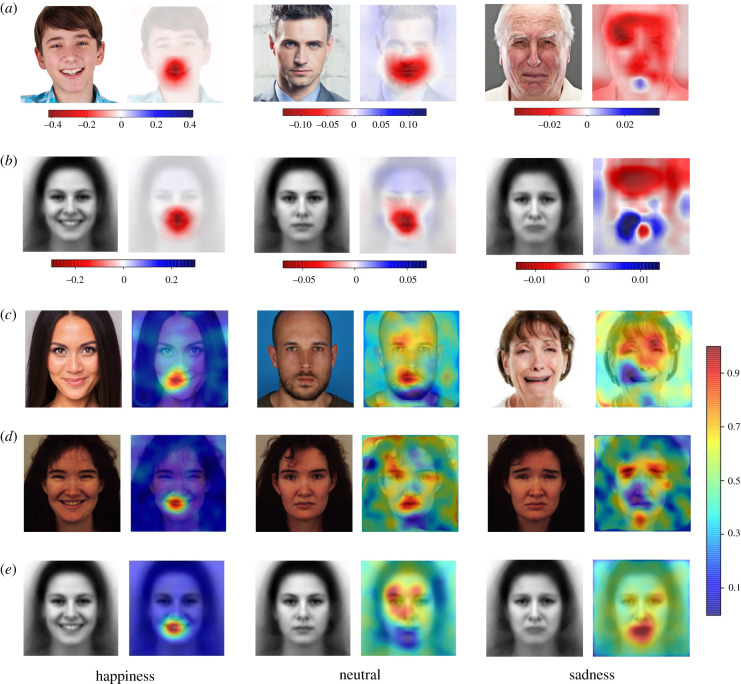
(*a*) Variation in the probabilistic classification score from classification baseline, obtained with the occlusion sensitivity analysis with three instances from the test dataset. The red areas indicate a reduction in the recognition ability of the model when a particular location is occluded, while the blue areas indicate the opposite. The colour bars indicate the appropriate probabilistic scale for each instance. (*b*) Average of the occlusion analysis in the KDEF dataset projected on the relevant AKDEF images. (*c*) Example of bubbles analysis on three subjects from the test dataset. (*d*) Example of bubbles analysis on a single subject from the KDEF dataset among the three categories. (*e*) Average bubbles result by category, projected on the relevant AKDEF dataset images (female AKDEF shown).

Expression discrimination from sparsely sampled faces, as during occlusion or bubble presentation, can reveal the spatial locations and therefore face features, that are more salient and probably affect emotional categorization. Overall, results from the occlusion analysis and bubble method are highly coherent with both datasets, thus documenting the robustness and reliability of the model (figure 7).

Regarding happy expressions, recognition accuracy drops drastically when the mouth is occluded, whereas the occlusion of other face areas has minimal impact on the model's performance. Symmetrically, revealing the mouth region in the bubble technique drives better discrimination performance than exposure of other face parts. Human psychophysics using the bubble technique have shown that in tasks requiring discrimination between happy and neutral facial expressions [120] or between all six basic emotional expressions [121], the mouth region provides the most important diagnostic information for the recognition of happy faces. The assignment of faces to happiness in our model, as well as in human observers, may also depend on appearance changes in the eye region [122]. However, the relevance of eyes in happy face recognition seems attributable to long-range influences from the changes in the mouth region and becomes informative only in specific tasks or when changes in and around the mouth region are diminished [123].

The above pattern is almost inverted for sad expressions. In fact, the view of the eyes and eyebrow region is most useful to the model for correct categorization of faces as sad, while the mouth becomes less salient. Neutral expressions fall midway, with the strength and reliability of the association between facial features and correct recognition depending more on a global scan path that includes both mouth and eye regions. Therefore, also in the present case our network model approximates the behavioural pattern of humans, who appear to extract and use the same small set of localized information specific to each expression for recognition [4,124,125].

## 4. Discussion

Compelling evidence is revealing a role of the SC in tasks and behaviours usually ascribed to the cortex, such as building multiple maps of the surrounding space, linking them to saliency in relation to individual needs or modulating attention and decision-making [19,21]. For example, orientation response tuning suggests that the SC is involved in early stages of contour perception and figure-ground segmentation and not simply in target selection for saccade generation. Moreover, several types of neurons in the superficial layers of macaque's SC respond very poorly to simple visual stimuli and their activation requires real objects or certain two-dimensional patterns [126].

Among the manifold functions the SC contributes to, it seems to possess the necessary infrastructure to conduct a value-based appraisal of emotional signals and then mediate approach and avoidance behaviours accordingly. Characterizing the SC's computational properties before further analyses on incoming input occurs in other downstream structures is paramount to gain an evolutionary understanding of the visual brain. In fact, the functions and circuitry centred on the SC are well conserved throughout vertebrates, predate the evolution of neocortex, and appear early during phylogenetic as well as ontogenetic development. For example, the SC is present in reptiles, birds and mammals, its neurogenesis is complete at birth, and its connections laid down by the first weeks of age [19,21,52]. However, its localization in the brain, dimensions, and the difficulty to ‘isolate’ it from the influence of other brain areas have limited our knowledge of its computational properties, especially in humans. In this context, a DNN imitating the neurobiological constraints of the retino-collicular system can swiftly provide a preliminary tool to perform ‘*in silico* neurophysiology’ [127], thereby promoting a milestone to furthering neuroscience investigation in the human and non-human primate brain.

Neural networks models offer a principled perspective to specifying mechanistic hypotheses on how sensory and cognitive functions may be carried out by real brains [100]. In fact, DNNs have found their place in the plurality of models available in neuroscience because they fulfil serval functions exceedingly well [128]. First, DNNs make falsifiable predictions that can be compared to specific brain systems in terms of detailed patterns of behaviours. This has led to the development and optimization of experimental designs for empirical inquiry in the real brain. Second, they contribute to exploring and generating new hypotheses through proof-of-principle demonstrations that create plausibility and motivate further research, especially in the absence of a fully fledged theory of how a cognitive function emerges from neural architectures. DNNs have made strides in elucidating principles of development and organization in the primary (geniculo-striate) cortical visual system, more specifically in the ventral stream [48,51]. However, a circuit-level DNN model that reproduces the architecture and neurobiological constraints of the retino-collicular system has never, to our knowledge, been devised yet.

The present study marks a first step towards the investigation with artificial neural networks of salient facial features based on expression perception carried out uniquely by the retino-collicular system. When the field of inquiry is in its infancy, and the understanding of a biological phenomenon is limited, as in the present case, the equivalence between DDNs and biological brains can be profitably understood mainly in the context of the behavioural outcomes they produce. Noteworthy, several similarities to human behaviour and primate neurophysiology emerged spontaneously in our model, or arose simply as a consequence of the artificial network learning to perform the task, rather than the modeller imposing parameters besides the neural architecture and the objective goal [46,47].

First, the model matched expression recognition accuracy of V1-damaged patients, who supposedly rely on the retino-collicular system for stimulus encoding. Face categorization was significantly lower than the one displayed by healthy observers, but still above chance and in the predicted accuracy range, including a remarkable correspondence with human data in the error patterns and confusion across categories [38,109,110].

Second, tuning to LSF and orientation selectivity emerged spontaneously in the model and were different from those developed by established geniculo-striate networks presented with the same gratings. The discrimination performances were in accordance with psychophysical evidence in human blindsight patients and are plausibly generated by the neurophysiological properties that the primate SC derives from the prevalent M and K retinal input over the P pathway [26,38,40]. This suggests that initial phases of facial encoding may not depend on the development of specialized cortical areas but seem to capitalize on phylogenetically ancient visual structures already existing in new-born primates and other lineages.

Third, the results on spontaneous tuning led to testing the impact of different approaches to image manipulation that can bias face processing towards M and P channel properties. DNN recognition of M-biased faces filtered to display only LSFs was not measurably altered compared to broad-band images, whereas the classification of P-biased expressions with HSF was almost at chance level. We also provide proof-of-principle that alternative methods to engage the M or P channels activate the human SC differentially in fMRI. Future fMRI studies could determine whether this increased SC activity to M-biased faces withstands larger samples than our two subjects, and if an additive difference between image manipulation and expressions emerges. In the interim, these findings provide initial evidence that adjusting the luminance and colour of image stimuli to unbalance M and P processing impacts SC activity, as predicted by our model.

Fourth, we explored the effects of different sources of uniform image noise and the assignment of saliency to specific image locations and facial features depending on emotional expressions. Results show that the mouth region (and therefore the smile) is crucial for categorizing happy expressions, and the eye area is most useful to recognize sadness [123]. These computations arguably reflect internal representational transformations instantiated by the DNN that progressively defines which features are more relevant to perform the task.

Clearly, the fact that our model of the retino-collicular system seems capable of expression discrimination and predicts several patterns of human and primate behaviours does not mean that other brain areas are not crucial for emotional processing, or that the final encoding is not the by-product of complex interaction at the system level [129]. The key issue is that the retino-collicular system can actively instantiate early emotional evaluation and pass it on to these other brain structures, contrary to the prevailing view [16].

Our circuit-level model of the retino-collicular system can be used to delve more deeply into understanding the development and organization of early visual processing for facial expressions and other stimulus categories. In fact, modelling can be heuristically influential and provide traction for new empirical testing of V1-independent vision in human and non-human primates. For example, responses to uniform image noise, or perturbations to stimulus attributes in the neural network can be the testing ground for experiments in humans and monkeys to sample new functional properties of the SC. The network can be used to compute a maximally exciting input image (MEI) that strongly activates specific layers or neurons in the model. This MEI can then be presented to humans or monkeys without V1, and the resulting neural response measured [130]. If the deep network captures the mapping from image features to neural response, the MEI should also excite the biological neurons. On the other side, histological and neurophysiological studies offer viable support to refine artificial models and interpret V1-independent vision centred on the SC. These are outstanding research questions for neuroscientists interested in exploring how many visual computations the retino-collicular system can account for, based on a bottom-up approach.

## Data Availability

The source code of the model, including scripts for evaluation, is available as electronic supplementary material [131].
